# 3D/4D Printing of Polymers: Fused Deposition Modelling (FDM), Selective Laser Sintering (SLS), and Stereolithography (SLA)

**DOI:** 10.3390/polym13183101

**Published:** 2021-09-15

**Authors:** Abishek Kafle, Eric Luis, Raman Silwal, Houwen Matthew Pan, Pratisthit Lal Shrestha, Anil Kumar Bastola

**Affiliations:** 1 Design Lab, Department of Mechanical Engineering, Kathmandu University, Dhulikhel 45200, Nepal; abishek.kafle@ku.edu.np (A.K.); ramansilwal24@gmail.com (R.S.); 2 Faculty of Medicine, Macau University of Science and Technology, Avenida Wai Long, Macau SAR, China; laguntureric@must.edu.mo; 3 Division of Chemistry and Biological Chemistry, School of Physical and Mathematical Sciences, Nanyang Technological University, 21 Nanyang Link, Singapore 637371, Singapore; matthew.pan@u.nus.edu; 4 Centre for Additive Manufacturing (CfAM), School of Engineering, University of Nottingham, Nottingham NG8 1BB, UK

**Keywords:** 3D printing, 4D printing, fused deposition modelling, selective laser sintering, stereolithography, polymers

## Abstract

Additive manufacturing (AM) or 3D printing is a digital manufacturing process and offers virtually limitless opportunities to develop structures/objects by tailoring material composition, processing conditions, and geometry technically at every point in an object. In this review, we present three different early adopted, however, widely used, polymer-based 3D printing processes; fused deposition modelling (FDM), selective laser sintering (SLS), and stereolithography (SLA) to create polymeric parts. The main aim of this review is to offer a comparative overview by correlating polymer material-process-properties for three different 3D printing techniques. Moreover, the advanced material-process requirements towards 4D printing via these print methods taking an example of magneto-active polymers is covered. Overall, this review highlights different aspects of these printing methods and serves as a guide to select a suitable print material and 3D print technique for the targeted polymeric material-based applications and also discusses the implementation practices towards 4D printing of polymer-based systems with a current state-of-the-art approach.

## 1. Introduction

3D printing or additive manufacturing (AM) is a digital manufacturing process, in which the materials are added layer by layer to create 3D objects directly from the computer-aided design (CAD) models [1,2,3,4,5,6,7,8]. 3D printing has gained significant popularity in the last two decades due to a number of appealing advantages such as the limitless design freedom and capability to produce low cost and multifunctional objects with highly delicate/complex structures in a short period of time [9]. For example, 3D printing of concrete materials possesses the potential to reduce construction waste by 30–60%, labour cost by 50–80%, and construction time by 50–70% [10,11]. Therefore, 3D printing has become a suitable manufacturing technique in both rapid prototyping as well as in various engineering fields such as mechanical engineering, civil engineering, aerospace, electronics, biomedical, etc. [5,6,9,12,13,14,15,16,17,18,19].

**Figure 1:**
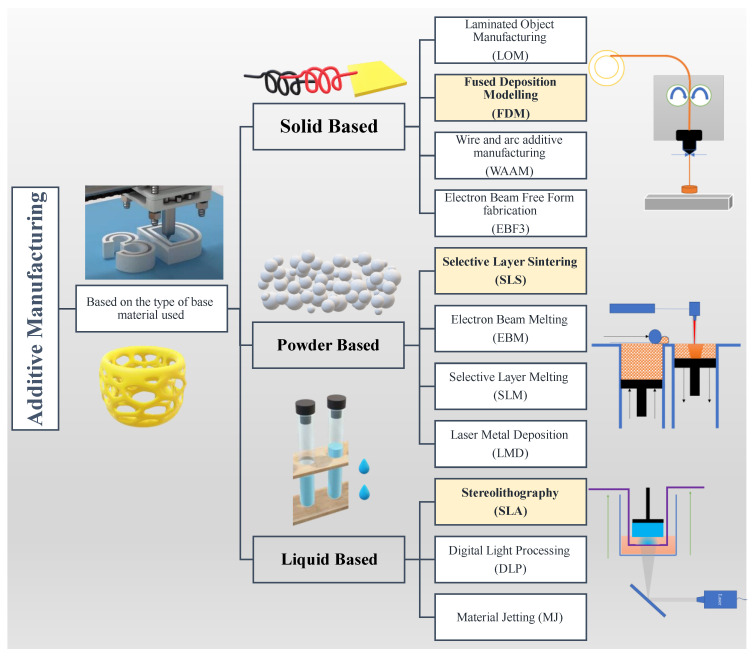
Classification of AM techniques based on the type of base materials used and the scope of the current review as highlighted (FDM, SLS, and SLA). The 3D printing image is taken from [72].

A variety of AM methods are available to 3D print a wide range of materials including metals [20,21,22,23], polymers [24,25,26,27,28,29], polymer composites [30,31,32,33], ceramics [34,35,36,37,38,39], and cement [40,41,42,43]. The ASTM (ISO/ASTM 52900:2015) has classified the range of AM processes into seven general categories. This classification is made on the basis of the fundamental principle of operation, and it includes material jetting, binder jetting, vat photopolymerization, powder bed fusion, material extrusion, direct energy deposition, and sheet lamination [5]. Furthermore, according to the type of base material used, AM can be grouped into three different categories i.e., solid-based, powder-based, and liquid-based (Figure 1). The solid-based AM is further classified into laminated object manufacturing (LOM), fused deposition modelling (FDM), wire and arc additive manufacturing (WAAM), and electron beam free form fabrication (EBF3). Powder-based additive manufacturing can be classified into selective laser sintering (SLS), electron beam melting (EBM), selective laser melting (SLM), and laser metal deposition (LMD). The liquid-based methods mostly include material jetting (MJ) and vat-based printing such as stereolithography (SLA) and digital light processing (DLP). We refer to these excellent review articles to get a comprehensive insight into the above-mentioned AM techniques, LOM [44,45,46], FDM [47,48,49,50,51,52], WAAM [53,54], EBF3 [55], SLS [56,57], EBM [58,59], SLM [39,59,60], LMD [61,62], SLA [63,64,65], DLP [66,67,68], and MJ [69,70,71].

A polymer is a substance or material consisting of very large molecules, or macromolecules, composed of many repeating subunits [73]. Polymers are one of the prominent materials in a number of different applications due to their wide range of mechanical, thermal, electrical, fire-resistant, and biocompatible properties. According to the Web of Science (accessed on 2 August 2021), more than 60% of AM studies are focused on polymer printing. The polymers can be 3D printed with all three (i.e., solid-based, powder-based, and liquid-based) AM techniques [74]. FDM is the most conventional and widely used solid-based 3D printing technique to create polymer parts. On the other hand, SLS is a prominent AM technique to produce polymer parts using polymeric powders as a base material, while a vat-based technique, SLA, is another widely used early adopted AM technique to create polymer parts by processing the polymeric liquid as a base material. The details of these print methods are discussed in Section 2.

Although there are a number of review articles available in the literature focusing on various aspects of polymer printing based on FDM [47,48,49,50,51,52,75,76,77], SLS [1,78,79,80,81], and SLA [82,83,84,85], to the best of the authors’ knowledge, a comprehensive study focusing on correlating the material-process-properties for these techniques is not available for both conventional 3D printing and emerging 4D printing techniques. In this article, we aim to provide the correlation of material-process-properties for these three most conventional yet widely adopted polymer-based 3D printing techniques; fused deposition modelling (FDM), selective laser sintering (SLS), and stereolithography (SLA). Furthermore, we also briefly cover how these methods are adopted towards the 4D printing (3D printing of smart materials) of polymer-based materials giving an example of 4D printing of magnetic field responsive polymers.

## 2. Printing Process

**Figure 2:**
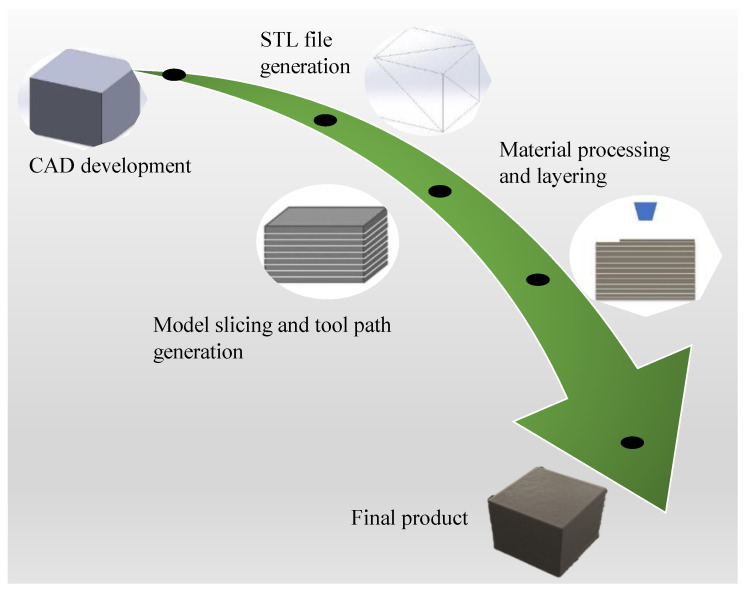
The workflow of the 3D printing process.

The fundamental process of 3D printing is the formation of parts by printing successive layers of materials that are formed on top of each other. The workflow of the 3D printing process is illustrated in Figure 2. Firstly, the CAD model of the object to be developed is created, then the standard tessellation language (.stl) file of the CAD is generated. The STL file creation process mainly converts the continuous geometry in the CAD file into small triangles [86]. The .stl file is then exported in a model slicing software which creates a tool path for the 3D printer. Here, the 3D model is translated to 2D slices that contain the information of cross-sections [87]. The 3D printer then starts the material processing and layering process. The final product is then taken out of the printer. There are a number of different factors in the printing process that determine the overall quality of the printed parts. For example, the .stl file can influence the overall quality of the printed objects. The finer the size of the triangles in .stl file, the better the printed object/part shape fidelity. The part orientation during the printing process is responsible for the mechanical properties while the environmental factors such as temperature and humidity also play the role on the overall quality of the final product [88].

Fused deposition modelling (FDM) also known as fused filament fabrication (FFF) is a process of depositing thermoplastic filaments layer by layer on a build platform [30,49,89,90,91,92,93,94,95]. The polymer filament is heated to a semi-solid state and deposited on the print bed or heated platform. The nozzle follows the path as of the final object in the given layer. For the next consequent layer, the platform moves one step lower, or the nozzle moves one step upward, and the material is extruded and again the nozzle follows the path of the object in the given layer. To generate the path that the nozzle follows, a slicer slices the model layer by layer and produces a G-code (computer numerical control programming language), which is followed by the nozzle in each layer. The height that the nozzle travels after each layer is the layer thickness of the model. The nozzle temperature, bed temperature, and layer height are the responsible parameters for the fractional behaviour of the 3D printed parts [96].

**Figure 3:**
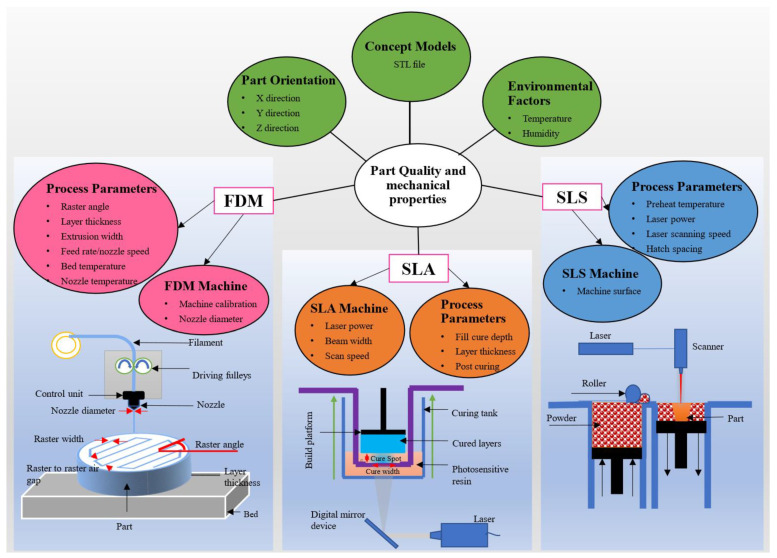
Overview of the process parameters of three different print methods: FDM (**left**), SLS (**right**), and SLA (**middle**).

Print parameters can be grouped as machine parameters and process parameters for each printing technique (Figure 3).

The machine parameters for FDM printing are bed calibration and nozzle diameter while the process parameters are nozzle temperature, bed temperature, extrusion width, and raster angle [52,97], see Figure 3 (left) for the schematic illustration. The bed calibration is one of the most important considerations. FDM is a contact print method as the nozzle is used to directly deposit the material layer by layer, therefore, the distance between the nozzle and bed should be at a standard distance and constant throughout the bed. An improper bed calibration leads to the uneven distance between the nozzle and the bed at two (or more) different points on the print platform/bed, which causes warpage and also leads to the printer hitting the bed and the prints. The diameter of the 3D printing nozzle can be changed/replaced which however impacts the part quality and production time. The use of a nozzle with a large hole diameter accelerates the part production time [98]. It has also been reported that increasing the nozzle diameter increases the part quality and mechanical properties in FDM 3D printing [99]. The other important parameter is the ambient temperature which causes part warpage. For example, PLA part warpage of about 50%, 30%, and 10% at 10 °C, 15 °C, and 20 °C (ambient print environment temperature) respectively, is reported [100]. The process parameters affecting part properties in FDM are raster angle, extrusion width, extrusion rate, bed temperature, nozzle temperature, and nozzle speed. The raster angle is the angle between the direction of the nozzle and the *x*-axis (or *y*-axis, depending on notation) of the printing platform [101]. The extrusion rate is the rate at which the filament is extruded from the nozzle onto the build platform. Bed temperature refers to the temperature of the build platform. The bed temperature is required to maintain the adhesion between the build platform and the print part and avoid warpage [102]. Nozzle temperature is the temperature at which the material is melted and extruded from the nozzle. It has a high influence on the mechanical properties and microstructure of the 3D printed parts [99]. For instance, the increase in relative density (from 89.9% to 92.8% for PEEK) with the increase in nozzle temperature from 370 °C to 390 °C is reported [103]. Nozzle speed is the speed at which the nozzle moves while depositing/printing the filament melt from the nozzle onto the build platform. It greatly influences the dimensional precision of the printed parts although print time is reduced. For example, the increase in wall thickness of ring-shaped design from 2.00 mm to 2.17 mm with the increase in nozzle speed from 30 mm/s to 90 mm/s is reported [104].

**Figure 4:**
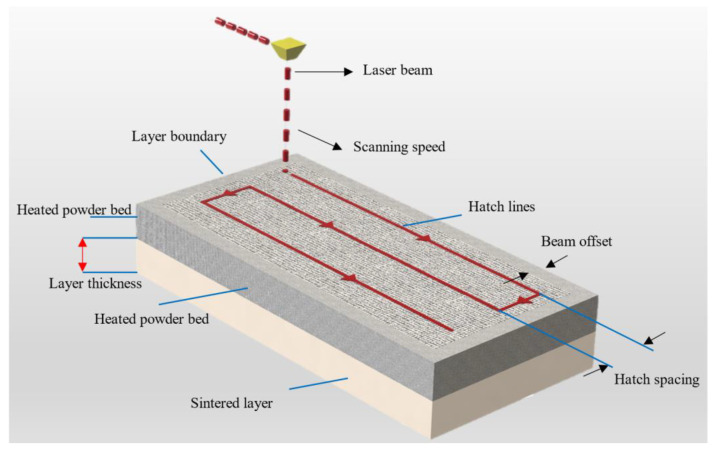
Process parameters for SLS printing, redrawn from [105].

Selective laser sintering (SLS), a variant of powder bed fusion and widely used AM technique, is a process used to produce objects from powdered materials using one or more lasers to selectively fuse the particles at the surface, layer upon layer, in an enclosed chamber [57,105,106,107,108]. The powders can be fused together with different particle binding mechanisms namely solid-state sintering, chemically induced binding, liquid phase sintering (partial melting), and full melting [109]. The working schematic of SLS is described in Figure 3 (right), also see Figure 4 for SLS process parameters. The printing system consists of a laser supply source, scanning system, roller, powder supply platform, and a sintering platform. Usually, the powders are fused by molecular diffusion under the influence of a high-power laser. After the first layer of powders is fused the sintering platform moves a step downwards and the next layer of powders are fused [110]. The process continues until the top layer of the final product is fused. The movement of the laser is determined again by the G-code generated from the slicer like in FDM. After the sintering process is completed, the un-sintered powder is removed, and the part is extracted from the platform.

The energy density (Equation (1)), in SLS, is the most vital parameter that is responsible for the overall process and part property. It is the amount of energy stored in a given system or region of space per unit volume.

(1) $$ED=\frac{P}{v.h}\;\times \;\frac{d}{h}$$

where *ED* is energy density, *P* is the laser power, *d* is laser beam diameter, *v* is scan velocity, and h is the hatch spacing. The hatch spacing, laser scanning speed, laser power, and preheat temperature are therefore determining process parameters responsible for the part properties of SLS printed objects [105,111,112], see Figure 4. Laser power is the input power set as the ratio of the total permissible power as per the requirement of a given material and layer thickness [113]. Laser scanning speed is the rate at which the laser beam is moved along the hatching or contour lines. It influences the maximum energy at a point of material and the total time required to complete a product [114]. Hatch spacing is also known as scan spacing is the distance between two consecutive laser beams. Preheating temperature is another important parameter that affects the part property in SLS. A powder that is not preheated requires a higher-power laser beam source to melt. Furthermore, higher preheating also reduces the temperature gradient between the sintered and un-sintered parts—contributing to the elimination of thermal stress and avoiding distortion [115].

Stereolithography apparatus (SLA), a vat-based and the early adopted AM technique, works on the process of 3D printing by using photopolymerization in which the photocurable resin is solidified through photopolymerization initiated by absorbing light [82,84,116,117,118,119]. Photopolymerization refers to a technique that uses rays of light to propagate a chain polymerization process which results in the photo-crosslinking of the pre-existing macromolecules [116]. The crosslinker is another component/material that links one polymer chain to another by the covalent or an ionic bond. The photopolymerization results in the solidification of a pattern inside the resin layer in order to hold the subsequent layers. A photoinitiator or photoinitiator system is required to convert photolytic energy into the reactive species (radical or cation) which can drive the chain growth via radical or cationic mechanism [116]. The measurement of attenuation of light by a chemical species at a given wavelength is given by the molar attenuation coefficient. The molar attenuation coefficient is a measurement of how strongly a chemical species attenuates light at a given wavelength. Typically, photoinitiators with molar attenuation coefficients at a short wavelength (UV < 400 nm) are used to initiate the photochemical reaction [120]. Using a computer-controlled laser beam, a pattern is illuminated on the surface of a resin. The area in the resin where the light beam strikes solidify. This principle is used repeatedly layer by layer to solidify the resin and form each layer of a product in SLA 3D printing. The thickness of the layer is controlled by the energy of the light source and exposure time [64].

The major process parameters that influence the quality of SLA printed parts are fill-cure depth, layer thickness, and post-curing, see Figure 3 (middle) for the illustration of the process parameters. The cure depth depends on the energy of the light being exposed to the resin. The energy is controlled by the laser power and the time the resin is being exposed to the light. The curing depth (C_d_) should be high enough to avoid excessive fabrication time. However, the curing depth must be low enough to avoid over polymerization resulting in the over-cured part with poor resolution. Curing depth is given by an equation based on the Beer–Lambert equation (Equation (2)):

(2) $$Cd_{\;}=Dp_{\;}log\frac{E}{E_{c}}$$

where D_p_ is the penetration depth (m), *E* is the light exposure (J m^−2^), and *E_c_* is the critical light exposure (J m^−2^) [121]. The wavelength of the laser light being used is another important consideration. The wavelength of the UV light reported in the literature is in the range of 300 nm to 400 nm [63]. Usually, in SLA, objects/parts need to be post-cured after printing. Post-curing is performed to enhance the mechanical properties of the printed objects/parts. For example, a post-curing time up to 60–90 min for SLA 3D printed dental parts such as crown and bridge materials is reported [122].

The printing parameters required for FDM, SLS, and SLA 3D printing are collectively summarized in Figure 3. The initial printing parameters such as quality of the .stl file, part orientation, and environmental factors are common printing process parameters in all three techniques. However, due to the variation in the structure formation technique, the printing process and a number of process parameters differ in each of these methods. The printing techniques can be chosen according to the requirement of the simplicity of printing, mechanical properties, printing time and layer resolution. For instance, SLA has the capability of printing high-resolution parts of up to 10 µm [33], while the minimum layer resolution of the SLS printed part is 20 µm [123] and the FDM only has the capability of printing high-resolution parts of up to 40 µm [124]. The value for the print resolution should be considered as a comparative guide only because the exponential growth of the 3D printing industry is continuously offering optimized versions of the 3D printers. The print resolution of some common commercially available 3D printers is listed in Table 1. Based on the data provided by the manufacturer, the print resolution up to 25 µm [125], 50 µm [126], 1 µm [127] for FDM, SLS, and SLA, respectively, is also claimed. On the other hand, in terms of the process simplicity in printing, the FDM is the most suitable because the process is as simple as heating the filament polymer to a semi-solid state and depositing it directly on the print bed. SLS is a comparatively complicated process among others, it requires the movement of two systems: roller and laser light. A list of commercially available FDM, SLS, and SLA 3D printers along with their material and print specifications is summarized in Table 1. This table provides an overview for selecting the desired 3D printer on the basis of the required print volume, material, and print process. From Table 1, it is also evident that the FDM provides a wide range of materials for 3D printing while SLA provides a high print resolution. Further discussion based on the material and print part properties is presented in Section 3 and Section 4.

## 3. Print Materials

The common print materials available for FDM, SLS, and SLA 3D printing are given in Table 2. The material for FDM and SLS are thermoplastic polymers. Due to the process requirements, for FDM, the material is in filament form, while for SLS, it is in powder form. The thermoplastic polymers can be classified into amorphous and semi-crystalline thermoplastic. Amorphous thermoplastic polymers have a glass transition temperature (T_g_) above which they soften and transform into a glassy state. They do not have a fixed melting temperature, while semi-crystalline polymers have a fixed glass transition temperature (T_g_) and melting temperature (T_m_). The melt viscosity of the semi-crystalline thermoplastics decreases with the increase in temperature above the melting temperature (T_m_)—allowing flowability [159]. Apart from the pure polymers, the use of several modified FDM filaments has also been reported for various applications such as printed electronics by incorporating different materials such as carbon-black, graphene, and copper [160], carbon nanotube incorporated filaments for textile [161], carbon nanotubes incorporated capacitive and piezoresistive actuators [162], etc. A few approaches of the modification of materials for FDM/SLS printing by giving an example of magnetic materials is discussed in Section 5.

The materials used in SLA are photosensitive thermoset polymers. Thermoset is also known as a thermosetting polymer and is a polymer that is obtained by irreversibly polymerizing/curing a soft solid or viscous liquid prepolymer (resin). The curing, also sometimes known as solidification or vulcanization or polymerization, is achieved via photopolymerization in the presence of UV light. An SLA resin usually contains several components including monomer/oligomer, diluent, chain transfer agent, and photoinitiator [84,163]. Monomers/oligomers are reactive prepolymers that are primarily responsible for the part properties after undergoing a polymerization reaction. Diluents are low-molecular weight, low-viscosity compounds used to modify the viscosity of a resin or enhance the solubility of a resin. A chain transfer agent is essential to modify the crosslinking agent while photoinitiator is necessary to trigger the photopolymerization. The widely used resins are polyester or polycarbonate or polyether-based polymers in SLA or the vat-based printings [24,84,163].

### 3.1. Material Requirements

**Figure 5:**
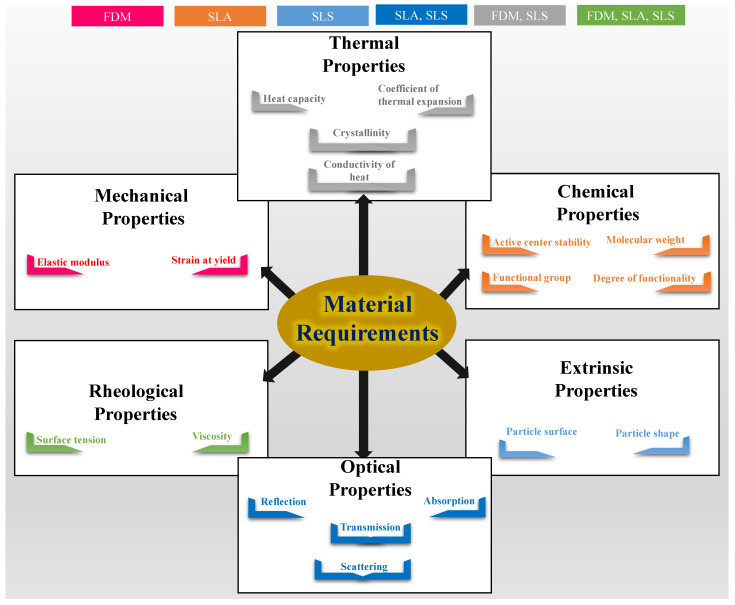
A pictographic summary of the various properties of print materials demanded for successful printability via FDM, SLS, and SLA 3D printing.

The material requirement majorly depends on the process of 3D printing. Figure 5 provides a pictorial summary of the material requirements for successful printability for all three print methods. Rheological properties are a common requirement of print materials in all three processes. It mostly includes the viscosity of the print material (polymer melt or resin). The thermal properties of the material include heat capacity, coefficient of thermal expansion, crystallinity, and conductivity of heat of the print materials. The thermal properties are an important consideration in FDM and SLS. On the other hand, in SLS and SLA, as both of these processes deals with absorption of energy through laser light, optical properties including reflection, absorption, transmission, and scattering are of utmost importance. In FDM, the mechanical properties of the filament including its elastic modulus and strain at yield is also considered. In SLS, extrinsic properties such as powder shape and powder surface are a considerable requirement. For SLA, chemical properties such as active centre stability, molecular weight, functional group, and degree of functionality of the resin play a key role. The details of material requirements and their significance are discussed in the following sections. Here, we first discuss individual process requirements of FDM, SLS, and SLA respectively and followed by the common requirements.

#### 3.1.1. Mechanical Properties

In FDM, the mechanical properties of thermoplastic filament are one of the major material properties to be understood. The column strength of the solid filament is significant for thermoplastic polymers [164]. In the printing process, the solid filament serves as a piston under compression; therefore, the column strength should be sufficient enough to avoid buckling between the driving pulley and the melt chamber [52,165]. The critical load for buckling is given by the formula derived from Euler’s buckling as given in Equation (3).

(3) $$P_{cr}=\frac{\pi^{2}Ed^{2}}{16L^{2}}$$

where *E* is the elastic modulus, *d* is the diameter, and *L* is the length of the solid filament between the driving pulley and the melt chamber [165]. The filaments should however be flexible enough to allow their spooling and despoiling during printing and thus maximum strain at yield is recommended to be about 5% [166,167].

#### 3.1.2. Extrinsic Properties

In SLS, particle shape, size, and distribution have a considerable influence on the overall flow behaviour and powder density. It is therefore an important consideration in SLS as it influences the thin, dense, and smooth layer of powders—influencing the quality of the produced part. The SLS powder particle distribution is reported to be between 20 μm and 80 μm [79], some particles have a higher size but mostly a d_50_ of around 60 μm [168]. The particle shape is required to be ideally spherical. Schmidt et al. [169] used the tensile strength to determine the powder flowability and concluded that the increase in powder flowability resulted in the decrease of tensile strength for the spherical particles.

#### 3.1.3. Chemical Properties

In SLA, the chemical properties of the resin are substantial material properties to be understood as the process comprises the photopolymerization completely driven by chemical reaction to convert the liquid into a solid object in the presence of UV light. Herein, curing kinetics is the most important consideration [170]. The curing kinetics is influenced by the degree of functionality, steric effect, and the stability of radical or cationic active centres, for more detail please refer to [171]. A moderate curing rate is required to enable the faster part fabrication and at the same time provide sufficient time for interlayer adhesion. A significant difference in static and dynamic properties has been reported for the curing time of 5 min in comparison to that of 25 and 30 min [172]. The curing degree increases with an increase in light intensity. For instance, the increase in curing degree from 3.1% to 87.7% with the increase in light intensity from 5 mW/cm^2^ to 40 mW/cm^2^ is reported [173]. The same study [173] also presents the influence of exposure time on the curing degree. The curing degree significantly increases from 26.85% to 70.98% for the same increase in light intensity. On the other hand, the curing degree only increases from 70.98% to 81.74% with the increase in exposure time from 3 s to 12 s.

#### 3.1.4. Thermal Properties

The thermal properties of the print material are highly influential material properties and need to be understood in-depth for successful printing via the SLS and FDM techniques.

**Figure 6:**
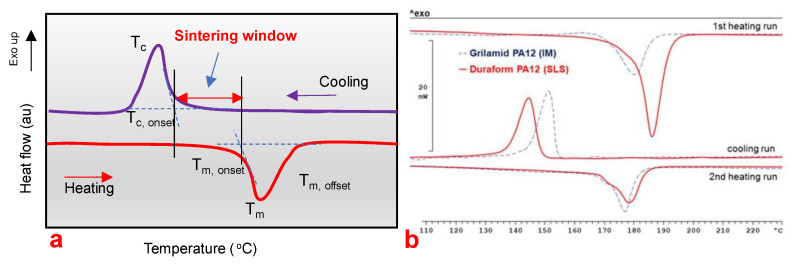
(**a**) Illustration of a dynamic DSC curve of a polymer. (**b**) Comparison of a commercial injection moulding PA12 grade and commercial PA12 for SLS processing, adapted from [168].

In SLS, the requirement of the laser energy intensity also depends on the temperature of the powder. The polymer powders are heated to a temperature close to the melting temperature for semi-crystalline powders and up to glass transition temperature for amorphous powders to lower the required laser energy and reduce the temperature gradient which also decreases the non-uniform shrinkage in the printed parts [174,175]. The preheating temperature should be close to melting temperature but should not be greater than the onset melting temperature to avoid premature melting of the powders. For general polymer materials, a preheating temperature 5–10 °C lower than the glass transition temperature is suggested [115]. Thus, the processing temperature must be precisely controlled between onset melting and onset crystallization temperature, and this metastable thermodynamic region is called the sintering window (Figure 6a) [176]. The sintering window can be characterized using a differential scanning calorimeter at a fixed heating and cooling rate, for example, 10 °C/min [168]. The sintering window, however, depends on the polymer being used. A wider sintering window is usually preferred in SLS. Figure 6b shows the thermo-analytical results (DSC measurement) of a commercial injection moulding PA12 grade in comparison with a commercial PA12 for SLS processing [168,177]. The stretch in the sintering window of SLS powders (red curves) can clearly be marked [168]. 

In FDM, the filaments are heated at a temperature a few degrees above the melting temperature at the nozzle. Reduced viscosity with increased temperature facilitates the polymer melt extrusion. The thermal properties of thermoplastic filament, moreover, influence the part shrinkage after the polymer melt deposition. The thermal properties include a coefficient of thermal expansion, heat capacity, heat conductivity and crystallinity of the polymer, please refer to these articles for more detail [178,179]. Similar to SLS, here, the thermal gradient leads to uneven shrinkage of printed parts [166]. For FDM, usually, the amorphous polymer filaments are favoured in comparison to semi-crystalline. The amorphous thermoplastics possess a low coefficient of thermal expansion—as a result, lower shrinkage, warpage, and distortion of the printed parts [180]. Another important consideration for FDM filaments is the printing temperature. This becomes an even more important consideration specifically for customized filaments with sensitive ingredients [181]. Usually, the printing temperature has to be above the melting point and should be always lower than the thermal degradation temperature of the print material. Thermogravimetric analysis (TGA) is used to characterize the filament material thermal stability by monitoring the weight change that occurs as the sample is heated at a constant rate. 

See Table 3 and Table 4 for a summary of the thermal properties of a few common thermoplastic filaments for FDM, and powders for SLS.

#### 3.1.5. Optical Properties

Optical properties are a key requirement of print material for its successful use in SLS and SLA 3D printing as materials absorb light in both processes.

The absorption of the energy from the laser source by the material is dependent on its optical properties. In SLS, a process involving the melting of polymer powders in presence of laser-generating heat energy, the polymer should be able to effectively absorb energy from the laser at a given wavelength. However, only a fraction of the energy is absorbed due to the laser reflection and refraction at the particle surface and transmission through the particles [182]. Most of the commercial SLS printers use CO_2_ lasers. This is because the polymer powders contain a C–H bond which absorbs the energy at the laser wavelength of 10.6 μm [183]. The thermoplastic powders after being exposed to the CO_2_ laser is transformed from an entropy elastic state to a viscous state [182]. To avoid warpage and shrinkage the laser power is desired to be on the lower side. With other printing parameters constant, the increase of laser power from 20 W to 35 W results in an increase in shrinkage from 2.34% to 2.60% and warping from 0.16 mm to 0.21 mm [34].

In SLA, the optical properties of the resin such as transmission, absorption, reflection, and scattering influence the curing depth. Detailed studies reporting the influence of these optical parameters on the cure depth (Equation (2)) are still lacking in the literature. It is recommended that the penetration depth is defined as the depth where laser irradiation is reduced by 1/e [24,184]. The absorption of light also highly depends on the concentration of the photoinitiator and the molar extinction coefficient of resin at the given light wavelength [185].

#### 3.1.6. Rheological Properties

The rheological properties of resin highly influence the SLA process and the melt rheology of powders to be used play an essential role in SLS.

The resins used in SLA must possess a melting temperature below the room temperature. The viscosity should ideally be around 1 Pas but it can range from 0.1 Pas for low-molecular-weight polymers to 10 Pas for high molecular weight polymers [186]. The lower viscosity allows the resin to be in a liquid state at the processing temperature enabling chain mobility. For the resins with higher viscosity, the resins can be processed at higher temperatures but this is limited only to formulations that are insensitive to heat [187].

In SLS, a powder with lower surface tension (γ) and lower zero viscosity (η_0_) is desired. It is because powder with lower surface tension has higher coalescence which can be sintered into parts of higher density and strength. The requirement of lower η_0_ is the reason behind the difficulty in sintering amorphous powders as the result leads to brittle and amorphous parts because of η_0_. In amorphous powder, the η_0_ is higher even above the glass transition temperature and thus a proper coalescence does not take place [79].

The investigation of the rheological properties of FDM filaments is well described in [188], in which applicability of the Filament Flow Index (FFI) is reported for a number of different filaments for FDM and suggested that the FFI technique can be considered to promptly characterize print filament. Elsewhere, to avoid buckling, the ratio of elastic modulus and viscosity of the FDM filament melts less than 3 × 10^5^ s^−1^ is recommended [166].

## 4. Properties of Printed Parts

The tensile and flexural mechanical properties of the FDM, SLS, and SLA printed parts are discussed.

**Figure 7:**
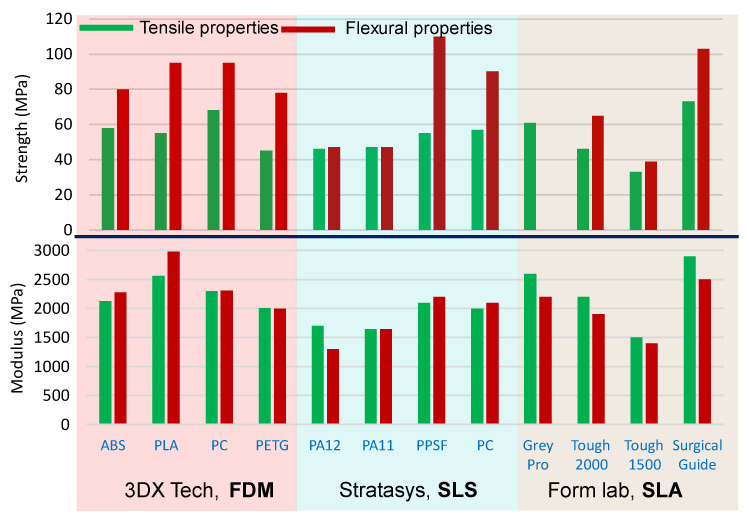
A graphical overview of the mechanical properties of 3D printed parts for a few commercially available materials, data are taken from the respective datasheet available on the supplier’s website. Tensile (green coloured) and flexural (red coloured) properties are plotted, the upper graph is strength, and the lower graph is the modulus of corresponding properties of FDM and SLS and SLA printed parts.

For commercially available print materials, the mechanical properties of the 3D printed parts are generally provided in the datasheet by most manufacturers. A pictographic overview of the mechanical properties based on the data provided by the manufacturer is given in Figure 7. In each 3D printing process, a range of variations in mechanical properties with different print materials can be noticed. This overview can be used to quickly screen the material and printing processes on the basis of the requirement of tensile and flexural properties. Modulus and ultimate strength are two key mechanical properties to be understood either in tensile or flexural loading. The data provided in the datasheet, however, might be based on a certain external condition favourable for generating optimum properties, therefore, these data should not be considered as a final property of the printed part for bespoke print conditions. Furthermore, it should be noted that selecting a print method and materials are also related to other properties such as fatigue properties, microstructure, stability, etc. [197,198,199,200,201]. In the following, the mechanical properties (tensile and flexural) of various print materials and printing processes reported by bespoke studies considering various print conditions are discussed. A summary of mechanical properties reported by a few bespoke studies is presented in Table 5 (tensile properties) and Table 6 (flexural properties). Anisotropy in mechanical properties is observed in all FDM, SLA, and SLS printed parts. This is primarily due to the fundamental process of the part production method in 3D printing (i.e., layer by layer addition of material). Typically, the mechanical properties of the 3D printed parts printed with sample build orientation parallel and perpendicular to the bed or print platform are found to be different, which signifies the anisotropy. Therefore, a part/object should be printed at an optimal orientation to achieve the best mechanical characteristics to meet the demand of the targeted application.

The variations in tensile and flexural strength with the change in build orientation and layer thickness in FDM, SLS, and SLA are discussed in the following section.

Please note that, apart from tensile test and flexural tests, nanoindentation is another prominent test for investigating mechanical properties such as modulus, hardness, and elasticity [202,203,204]. The biggest advantage of the nanoindentation test for the 3D printed part, compared to conventional tests, is that this test method can be used to study the localized anisotropy at various locations on the printed surface with minimum destruction limited to the surface of the material.

### 4.1. Tensile Properties

Tensile properties are used to study the behaviour of a material under the action of tensile loads. The tensile properties of a few standard materials available for FDM, SLA, and SLS 3D printing are given in Table 5. From the table, it can be concluded that the tensile properties of the polymers highly depend on the material, build orientation, and layer thickness. In FDM, the tensile properties of PLA and ABS have been most prominently studied while the properties of high-performance polymers like PEEK are also available. In SLS, the most widely used polymer powder is PA12 and its mechanical properties are widely studied. On the other hand, in SLA, the material depends on the manufacturer and the application for which it has to be used. The printing parameters such as laser power and bed temperature are varied based on the material being used while materials are printed with various layer thicknesses to alter the printing time which also alters the part strength. In most of the studies, the ISO 527 and ASTM D638 test standards have been used to determine the behaviour of the 3D printed parts under the influence of tensile loading.

**Figure 8:**
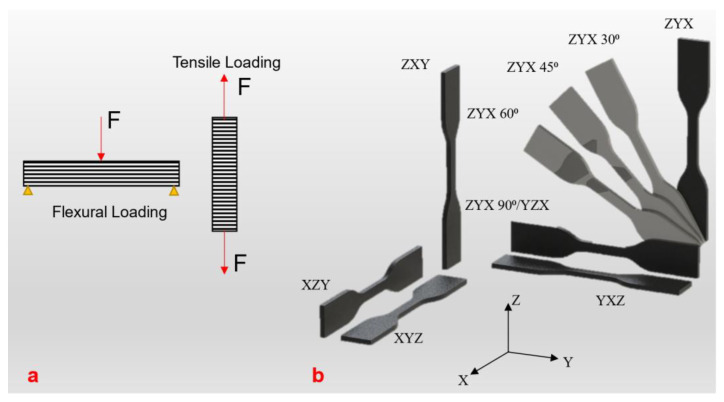
(**a**) Illustration of flexural and tensile loading and (**b**) definition of specimen orientation, redrawn from [16].

The tensile properties of FDM 3D printed parts have been well studied [205,206,207]. Printing can be performed at various orientations as illustrated in Figure 8 and the mechanical properties, therefore, are influenced by the print orientations. One common finding is that the tensile strength/modulus, when the load is applied in the longitudinal direction, is higher than applying load along the build direction; this is simply due to weak interlayer bonding of the printed parts [208]. Another factor contributing to the tensile strength of the FDM printed part is the raster angle. For instance, the ultimate strength, for PLA, obtained for a raster angle of 45° is higher compared to the raster angles of 0° and 90° [209]. For materials such as PEEK and ABS, the tensile strength of the printed material for raster angles of 0° and 90° was comparable to one another while the raster angle of 45° yielded a considerably lower amount of tensile strength [210,211,212]. The amount of material that has been deposited on each layer also affects the mechanical properties of FDM printed parts. The tensile strength increases linearly with the layer thickness when specimens are printed in the z-direction [213]. A study by Chacón et al. [213] can be consulted to get a comprehensive summary of the effect of process parameters on mechanical properties of FDM printed PLA and their optimal selection.

There is also a large disparity of mechanical properties for SLS 3D printed parts due to the dependence of various parameters on local process conditions [112]. This leads to properties such as modulus/stiffness and strength being highest along the print direction [214]. The parts built with orientations parallel to the direction of the laser exhibit the highest strength and modulus values while the samples built in the *z*-axis orientation possess the lowest strength and modulus. For example, the difference of 9.4% in strength and 7% in flexural modulus for these different build orientations is reported [215]. Furthermore, the specimens with the 60° raster angle exhibited the highest tensile strength when compared to the sample printed in 0°, 15°, 30°, 45°, 60°, 75°, and 90° orientations [216], unlike in FDM in which a 45° orientation showed the highest strength [209].

The tensile properties of a commercial photocurable resin (commercially available/manufacturer’s grade) have also been widely studied [217,218]. The tensile strength of various build orientations which include flat, and edge are widely reported [217,219,220]. In SLA, flat is similar to x and the edge is similar to y as in FDM printing. Furthermore, each build orientation had sub-orientations of 0°, 45°, and 90°, as in other print methods. Specimens with edge build orientation display higher tensile strength compared to the specimens with flat build orientation. In sub-build orientation, 45° orientations have slightly better properties than the 0° and 90° sub-build orientations in both cases [221]. Build orientation has much less impact on tensile strength when compared to layer thickness [216]. Tensile strength increases when the layer thickness increases while the flexural strength decreases [216]. The increase in tensile strength as layer thickness increases is due to better connection by polymerization of the new layer with the prior layer [74].

### 4.2. Flexural Properties

Flexural properties are used to determine the behaviour of a material under the action of bending loads. The flexural properties of 3D printed parts have not been studied as widely as the tensile properties. However, there are many studies that have given the flexural properties of some popular polymers of each 3D printing process. Table 6 is the compilation of flexural properties of various polymers fabricated using FDM, SLS, and SLA. The 3-point bending test is used in all of these studies. ASTM D790 is the most commonly used standard test method for the 3-point bending test. Like the tensile properties, the print parameters such as raster angle, part orientation, and layer thickness have a direct impact on the flexural properties of the 3D printed specimen.

In FDM, variation in mechanical properties with varying orientation and layer thickness can be distinctly observed. Layer thickness has the most significant effect on flexural strength. The increase in layer thickness is found to have an increment in flexural strength. For instance, a study focused on flexural strength of the specimen at different layer thicknesses ranging from 0.1 mm to 0.5 mm, reported a maximum flexural strength (59.6 MPa) at 0.5 mm layer thickness and minimum flexural strength (43.6 MPa) at 0.1 mm [222]. The raster angle also has significance on the flexural property [211]. Again, like in tensile loading, the PLA parts with a 90° raster angle showed the least resistance while the 45° orientation showed the highest resistance [209].

In SLS, the flexural strength is again influenced by bed temperature, laser watt power, scan speed, and scan spacing. For instance, the increase in flexural strength with an increase in laser power from 28 W to 36 W and a decrease in flexural strength with an increase in scan speed from 2500 to 4500 mm/s is reported [223]. In the case of the scan spacing, an initial decrease with an increase in scan spacing from 0.25 to 0.35 mm, then a marginal increase from 0.35 to 0.45 mm is reported [223]. Print orientation also has a notable influence on the flexural strength of SLS printed parts. For example, a maximum flexural strength at 0° (59.23 MPa) followed by at 45° (46.25 MPa) and minimum at 90° (19.89 MPa) is reported [224].

In SLA, the build orientation is the main factor influencing flexural strength. The specimen printed with a layer orientation parallel to the axial load is reported to have superior flexural strength and flexural modulus [88]. For example, maximum flexural strength was found at 90° (135.69 MPa), compared to 45° (130.73 MPa) and 0° (117.48 MPa) at ZXY orientation [88].

In Table 5 and Table 6, the findings of a number of different studies are presented for the comparison of mechanical properties under the action of tensile and flexural loading of FDM, SLS, and SLA printed parts. The tabular summary can be referred to screen and select a suitable print process and material to meet the requirement of the targeted application in terms of the mechanical properties.

## 5. Towards Magneto-Active 4D Printing

**Figure 9:**
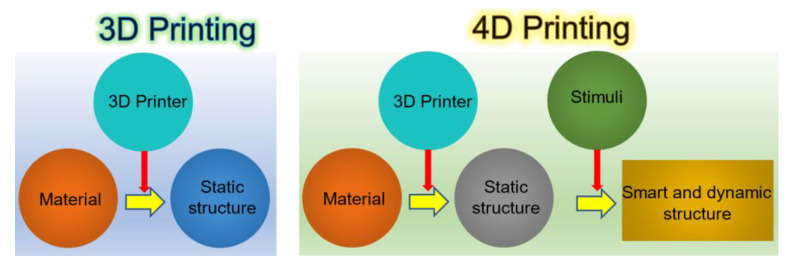
3D printing and 4D printing and their basic differences, redrawn from [259].

The 3D printing of smart materials is regarded as 4D printing (4DP) since it is first introduced in 2013 [234]. Smart materials can respond to external stimuli such as heat, pH, magnetic/electric field, etc. [235,236,237,238,239,240]. As illustrated in Figure 9, 4D printing essentially means the 3D printing of smart materials. The 4D printed structures can change their physical/chemical properties, for example, stiffness, density, etc., and demonstrate various phenomena such as shape memory effects and shape-shifting [235,236,237,238,241,242,243,244]. The shape memory effect is a phenomenon where a system/structure can remember a certain shape and could be switched from one to another shape (original to programmed shape) in a controlled way in the presence of external stimuli. Shape-shifting is a phenomenon where a system/structure can shift its shape from one to another when triggered by external stimuli. Here, we briefly cover the process and the material requirements to develop polymer-based 4D structures via FDM, SLS, and SLA printing. Our particular focus is magnetic field triggered systems.

The printing materials (resin/powder/filament) must have a magnetic field responsive element(s)/component(s) (typically known as fillers) to be triggered by an external magnetic field to demonstrate the 4D effect. Therefore, the first essential step to developing 4D structures via 3DP is to modify the printing materials by incorporating active components. The widely used magneto-active filler materials are carbonyl iron powders (CIPs), Iron (II, III) oxides, and Fe-Nd-B micro/nanoparticles [245,246,247,248]. All these magnetic fillers are yet to be implemented in all 4D printing methods (considered here) mostly due to the filler size. There are, however, other printing methods such as direct ink write (DIW) where nano to micron-sized fillers have been used successfully to a greater extent [236,249,250,251]. The filler materials should facilitate the re-extrudability of composite filaments for FDM [252,253], production of composite or surface decorated (with nanofillers) micro powders for SLS [254], and high stability in the liquid resin for SLA/DLP [255,256,257]. Please note that the pure SLA here is modified to its variants, to direct laser processing (DLP) or micro-continuous liquid interface production (μCLIP) or two-photon polymerization (2PP). All variants, however, are based on the light-mediated conversion of liquid resin [24,184,220,258].

**Figure 10:**
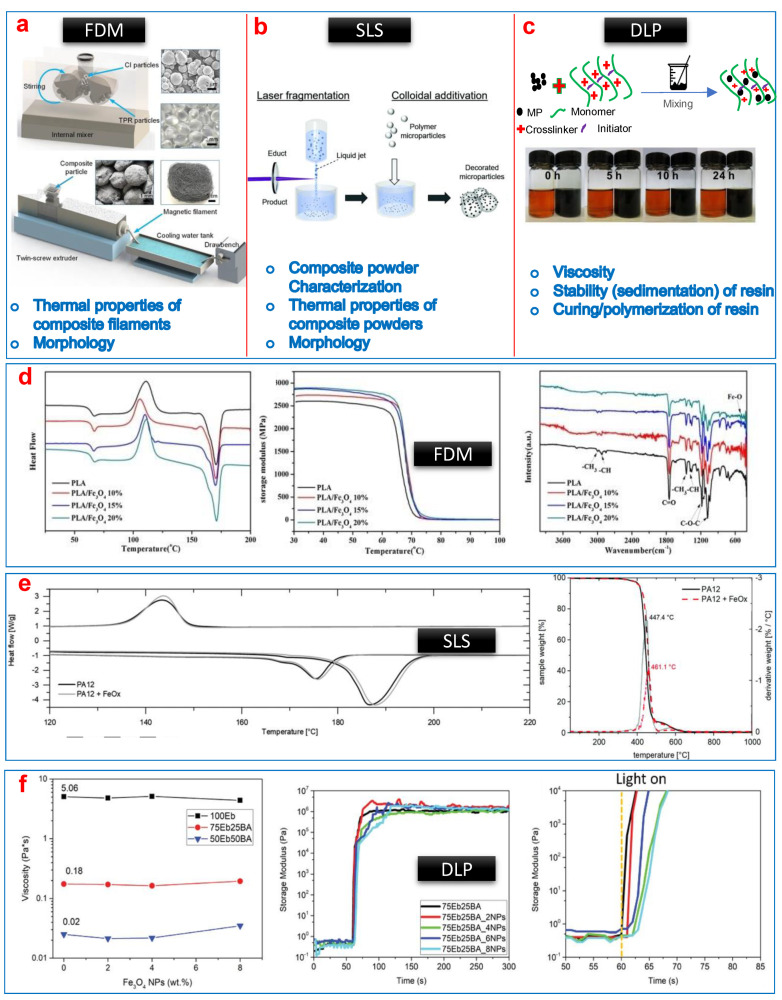
Material modification and requirement to develop magneto-active 4D structures via FDM, SLS, and SLA techniques. (**a**) Composite filament formation method (adapted from [253]), (**b**) composite powder formation method (adapted from [254]), (**c**) composite ink formation method (adapted from [256]), (**d**) examples of thermal and chemical properties of modified PLA-iron oxide composite filaments for FDM (adapted from [265]), (**e**) examples of thermal properties of modified PA12-iron oxide composite powder for SLS (adapted from [254]), and (**f**) rheological properties of acrylate iron oxide composite ink for DLP (adapted from [255]).

Figure 10 collectively shows material modification methods and key material properties of modified materials to be understood for all three different printing techniques. The main aim is to produce a composite filament with homogenously distributed fillers for FDM, composite powders with homogenously decorated/distributed fillers for SLS, and composite colloidal inks with homogenously dispersed/suspended fillers for SLA variants. 

Modifications of filaments for FDM include adding the filler to produce a homogenous mixture of the host material and magnetic fillers and the re-extrusion of composite filaments for printing. For instance, the original filament material (i.e., thermoplastic rubber) was heated to 70 °C to soften the surface then the magnetic fillers (i.e., CIPs) were added and mixed thoroughly. After that, a twin extruder was used to produce a composite filament [253]. In another study, PCL or TPU were mechanically mixed with CIPs first and then hot melted (at 200 °C) within the extruder and the composite filament was extruded [252]. Likewise, a composite filament of Fe_3_O_4_ nanoparticles and PLA was produced via melt compounding [260]. The processing factors such as melting, mixing, homogenization, granulation of compounds, and viscosity for extrusion play a vital role to produce composite filaments and there are a number of other different studies where the influence and optimization of these parameters are well reported, see Dohmen et al. [261], and others [262,263,264]. After the extrusion, cross-sectional morphology must be studied to investigate the homogeneity of the filler-matrix system. More importantly, the thermal properties of the composite filament need to be understood in detail for successful printing via FDM. For example, it has been reported that thermal properties (DSC and TGA thermographs) of the modified PLA with Fe_3_O_4_ nanofillers do not get altered significantly compared to virgin PLA (Figure 10d), hence the printing conditions (nozzle temperature, feed rate, print speed and so on) are altered to only a slighter extent [265], however, optimization of print parameters is still needed.

SLS printing sinters micron-sized powders to produce a part. In order to demonstrate the 4D effect, again the raw material (powders, e.g., PA12) should be modified by incorporating fillers. Compared to FDM and SLA (and its variants), there are very few studies where SLS is implemented to develop 4D structures of magneto-active polymers. To develop composite powders for SLS, the process is well reported in a recent article [254], wherein a novel method called nano-additivation is used. A colloid of magnetic fillers was formed first and then such magnetic particle-based colloid is laser fragmented by irradiating a laser light. The uniformly developed colloid is mixed with PA12 polymer to develop composite polymer powders for SLS (Figure 10b). Thereafter, the thermal material characteristics of nano-additively developed composite PA12 are studied using DSC and TGA methods. To print magnetic parts using such composites, all the process parameters such as temperature, laser output power, scan speed, hatch distance and energy density are studied in detail and optimized. It is reported that the thermal behaviour of the surface functionalized PA12 is similar to that of virgin PA12 powders (Figure 10e). In another very recent study (2021), micron-sized fillers (Nd-Fe-B) are used together with TPU [266]. Therein, composite is prepared just by blending the polymer and fillers, silica nanoparticles are added to improve the flowability. The mixing process is conducted just for 3 min at a rate of 600 rpm. Again, investigation of morphological, thermal, and mechanical properties of TPU/Nd-Fe-B composite is reported. The tensile strength of composites is found to be decreased compared to virgin TPU [266], while thermal properties remained similar to virgin TPU.

In SLA and its variants, which are resin-based systems, photopolymerization governs the conversion of liquid into a solid object in the presence of UV light (Figure 10c). Photopolymerization is a multi-stage and dynamic process therefore researchers have adopted different types of laboratory-based monomers/oligomers or copolymers to produce composite inks as a starting material. Various acrylate-based monomers/oligomers such as urethane acrylate with CIPs [267], with Nd-Fe-B [257], and with Fe oxides [255], and polyethene diacrylate with Fe_2_O_3_ [268] are reported. The development of composite ink is the mixing of different components (monomer, crosslinker, initiator), magnetic fillers and additives to produce a homogenous and stable ink [269]. Usually, the mixing procedure includes mechanical mixing followed by sonication. For ink, the essential material properties or parameters are resin viscosity, stability/sedimentation of fillers, and cure depth/penetration depth. The ink material properties highly depend on filler concentration and size (micro/nano) [256]. In the process, parameters such as exposer time per projection, layer height, waiting time before exposure, and influence of additives must be investigated and optimized [255]. For instance, viscosity and storage modulus (before and after exposer to UV light) of butyl acrylate-Fe_2_O_3_ composite inks are required to be very similar to that of virgin butyl acrylate resin (Figure 10f) [255], which defines the limit of the filler loading. Moreover, sedimentation is another major concern if micron-sized fillers are used [256]. There are a number of different studies where both materials and processes for vat-based printing of composite inks are studied in detail, refer to [255,256,269,270,271].

**Figure 11:**
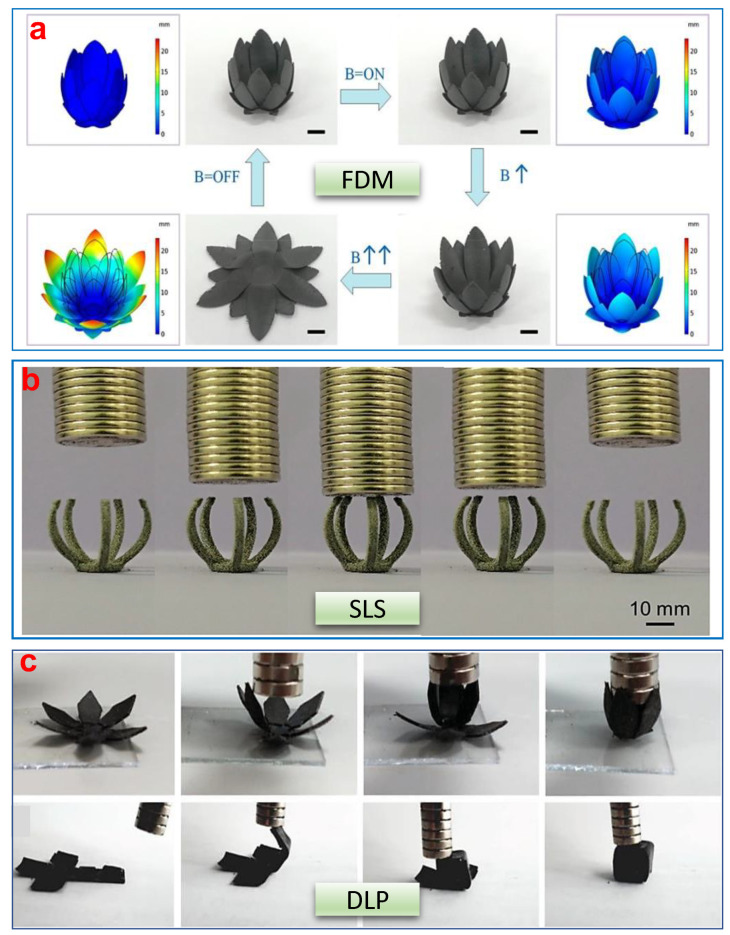
Examples of shape-morphing phenomenon demonstrated by 4D printed magnetic structures. (**a**) 4D effect of the flower-like biomimetic magnetic actuator under an external magnetic field, produced via FDM printing [253], (**b**) 4D effect of a gripper under an external magnetic field, produced via SLS printing [266], and (**c**) 4D effect of flower-like structure and folding of 2D to the 3D structure under an external magnetic field, produced via DLP printing [255].

A few typical examples of 4D printed structures of magneto-active polymers using FDM, SLS, and SLA (or its variants) are given in Figure 11. Development of a flower-like structure is one common practice to demonstrate the 4D effect, where such flower-like structures can blossom/open or close in the presence/absence of an external magnetic field. FDM and SLS produce a more rigid structure while resin-based systems (SLA variants) can produce flexible to rigid structures based on the varieties of tailored polymers because of the greater flexibility to control the polymer network. Magneto-active polymeric struc-tures possess a huge potential to exploit in a number of different applications such as in soft robotics and in the biomedical field, where shape-shifting or shape morphing is highly desired [237,238].

## 6. Concluding Remarks

AM is transforming the manufacturing industry with the ability to produce geometrically simple to highly complex and delicate structures. With a variety of 3D printing processes available for a wide range of materials, 3D printing has been extensively adopted in a number of different fields including but are not limited to mechanical engineering, civil engineering, aerospace, electronics, and biomedical. In this review, various aspects of the three most conventional, on the other hand, extensively adopted, 3D printing processes i.e., FDM, SLS, and SLA have been discussed. Correlation of three different aspects, materials, processes, and properties, for these polymer 3D printing techniques is presented. Each of these processes requires materials in a unique form which are filament, powder, and liquid for FDM, SLS, and SLA respectively. Although the fundamental of developing the product layer by layer remains the same, each method has a unique process and parameters of manufacturing to consider. A few process parameters such as CAD design of the model (.stl file), external environmental conditions, and the fundaments of 3D printing are similar in all the processes.

In order to select a 3D printing process for a specific application the requirements of print materials, properties of printed parts, the simplicity of printing, printing time, and layer resolution are essential factors to be considered (Table 7). For instance, SLA has the capability of printing high-resolution parts of up to 10 μm [33], while the minimum layer resolution of the SLS printed part is 20 µm [123]and the FDM has the capability of printing high-resolution parts of up to 40 µm [124]. On the other hand, in terms of the simplicity in printing, FDM is the most suitable technique because the process is as simple as heating the polymer filament to a semi-solid state and depositing it on the print bed. SLS is a comparatively complicated process among others, it requires the movement of two systems: roller and laser light. FDM 3D printers are the most economical and widely available while SLS 3D printers are the expensive ones. According to a recent study [272], a reasonable result in terms of accuracy can be achieved with all print methods; however, the preference should be more based on the print material, the intended application, and the budget. For example, dimensional accuracy and precision of 50 mandibular samples produced from various techniques show that the highest accuracy was found for SLS (0.11 ± 0.016 mm), followed by FDM (0.16 ± 0.009 mm) and SLA (0.45 ± 0.044 mm) [272]. In terms of print time, in their study, SLS (~ 48 min) is the fastest followed by FDM (~2 h and 40 min) and the SLA (~ 5 h 16 min plus post-processing ~15 min). On the other hand, in terms of cost, the SLS printer has the highest purchase price (e.g., EOSINT P 385 ~ USD 150,000), followed by the FDM printer (e.g., Ultimaker 3 Ext. ~USD 4000) and the SLA printer (e.g., Form 2 ~USD 3500), please note that the prices are estimates and may vary by reseller or country [272]. All in all, the FDM, being the simplest printing method, always marks higher in different aspects and thus is a highly reliable option in a number of different applications.

For each process, a wide range of materials are commercially available. For FDM, ABS, and PLA are the most prominently used thermoplastic filaments while, for SLS, PA12 powders are most widely used. For SLA, standard resins with unique formulations for different applications are developed by the manufacturers and there are a number of unique photocurable formulations based on polyester, polycarbonate, and polyether polymers, developed in the research laboratories. Although traditional SLA offers high lateral resolution compared to FDM and SLS, it is inadequately slow for large objects, therefore, a number of variants of vat-based techniques such as digital light processing (DLP), continuous liquid interface production (CLIP), and two-photon polymerization (2PP) have emerged to facilitate the printing time as well as resolution (2PP provides resolutions in the nm range).

4D printing, on the other hand, offers developing highly innovative and sophisticated devices/structures for various applications such as in soft robotics and the biomedical field. As reviewed in this work, FDM and SLA (mostly its variants) are increasingly adopted 4D printing techniques compared to SLS owing to their facile process and appropriateness for a variety of materials. The fundamental question/challenge of 4DP is how to modify the print materials (ink/resin/powder) as the well-established or commercially available materials cannot directly be used. The addition of various stimuli-active filler materials such as magneto-active, electro-active, and so on are to be incorporated successfully. Tailoring material properties, as well as optimization of the 3D print methods, are different from each other, i.e., for FDM, SLS, and SLA. In order to make printable materials as well as to meet the requirement of the targeted 4D application, a detailed investigation of the print materials and print processes is necessary. 4D printing is a highly interdisciplinary field and the expertise of material modification, process, and characteristics of targeted 4D effect is required for successful printing.

The material-process requirements discussed here in this review for a magneto-active polymer-based system remains similar for other active systems to develop other stimuli-active or even multi-stimuli-active 4D structures. It is believed that the correlation of material-process-properties of conventional polymeric material-based 3D printing together with an example of 4D printing provides a methodical guideline for 3D/4D printing of polymer materials using FDM, SLS, and SLA (or its variants).

## Figures and Tables

**Table 1:** A directory of commercially available industrial-grade FDM, SLS, and SLA 3D printers and their specifications, information is collected from the supplier’s website. The green highlight indicates the print material best suitable for the given printer according to the supplier. For SLA, the materials are categorized based on their type/specific properties as claimed by the supplier.

AM Technology	Name	Dimension of Printer (mm)	Print Volume (cm^3^)	Layer Thickness (mm)	Available Material/Type					Ref.
FDM	Stratasys F900	914 × 609 × 914	508,756.16	0.127–0.5	PLA	ABS	PEEK	Nylon	ULTEM	[128]
	Essentium HSE 280i HT	695 × 495 × 600	206,415	0.1–0.55	PLA	ABS	PEEK	Nylon	ULTEM	[129]
	CreatBot PEEK-300	300 × 300 × 400	36,000	0.04–0.4	PLA	ABS	PEEK	Nylon	ULTEM	[130]
	Anisoprint ProM IS 500	600 × 420 × 300	75,600	0.06	PLA	ABS	PEEK	Nylon	ULTEM	[131]
	3DGence F420	380 × 380 × 420	60,648	0.05	PLA	ABS	PEEK	Nylon	ULTEM	[132]
	Roboze Argo 500	500 × 500 × 500	125,000	0.025–0.2	PLA	ABS	PEEK	Nylon	ULTEM	[125]
	WASP 4070 Tech	400 × 400 × 700	112,000	0.1	PLA	ABS	PEEK	Nylon	ULTEM	[133]
	Cincinnati MAAM	1050 × 1015 × 1015	1,081,736.25	0.2	PLA	ABS	PEEK	Nylon	ULTEM	[134]
	Tractus 3D T850P	280 × 280 × 400	31,360	0.01–0.8	PLA	ABS	PEEK	Nylon	ULTEM	[135]
	AON-M2+	450 × 450 × 640	129,600	0.05–0.5	PLA	ABS	PEEK	Nylon	ULTEM	[136]
	Kumovis R1	180 × 180 × 150	4860	0.1–0.4	PLA	ABS	PEEK	Nylon	ULTEM	[137]
	Ultimaker S5	330 × 340 × 300	33,660	0.02–0.25	PLA	ABS	PEEK	Nylon	ULTEM	
SLS	Sintratec Kit	100 × 100 × 100	1000	0.05–0.15	PA 12	PA 11	TPU	TPE	PP	[138]
	Red Rock 3D	180 × 180 × 180	5832	0.1	PA 12	PA 11	TPU	TPE	PP	[139]
	Sinterit Lisa Pro	110 × 160 × 245	4312	0.05	PA 12	PA 11	TPU	TPE	PP	[140]
	Formlabs Fuse 1	165 × 165 × 300	8167.5	0.1	PA 12	PA 11	TPU	TPE	PP	[141]
	Sintratec S2	⌀160 × 400	8038.4	0.1	PA 12	PA 11	TPU	TPE	PP	[142]
	Sharebot SnowWhite 2	100 × 100 × 100	1000	0.05	PA 12	PA 11	TPU	TPE	PP	[143]
	Wematter Gravity	300 × 300 × 300	27,000	0.1	PA 12	PA 11	TPU	TPE	PP	[144]
	XYZ printing MfgPro230 xS	230 × 230 × 230	12,167	0.08–0.2	PA 12	PA 11	TPU	TPE	PP	[145]
	Nexa3D QLS350	350 × 350 × 400	49,000	0.05–0.2	PA 12	PA 11	TPU	TPE	PP	[146]
	Shining 3D EP-P3850	380 × 380 × 500	72,200	0.08–0.3	PA 12	PA 11	TPU	TPE	PP	[147]
	Prodways Promaker P1000	300 × 300 × 360	32,400	0.06–0.12	PA 12	PA 11	TPU	TPE	PP	[148]
	EOS Formiga P 110 Velocis	200 × 250 × 330	16,500	0.06–0.12	PA 12	PA 11	TPU	TPE	PP	[149]
	3D Systems ProX SLS 6100	381 × 330 × 460	57,835.8	0.08–0.15	PA 12	PA 11	TPU	TPE	PP	[150]
	Farsoon eForm	250 × 250 × 320	20,000	0.06–0.3	PA 12	PA 11	TPU	TPE	PP	[151]
SLA	Nyomo’s Minny	44 × 28 × 70	86.24	0.01	Standard resin	Castable	Bio compatible	Flexible	Clear	[152]
	Asiga’s Pico 2	51 × 32 × 76	124.032	0.001	Standard resin	Castable	Bio compatible	Flexible	Clear	[127]
	XYZprinting’s Nobel 1.0 A	128 × 128 × 200	3276.8	0.025–0.1	Standard resin	Castable	Bio compatible	Flexible	Clear	[153]
	Formlabs Form 2	145 × 145 × 175	3679.375	0.025–0.2	Standard resin	Castable	Bio compatible	Flexible	Clear	[154]
	Photocentric’s Liquid Crystal	121 × 68 × 160	1316.48	0.05	Standard resin	Castable	Bio compatible	Flexible	Clear	[155]
	Nexa3D’s the NXV	220 × 120 × 380	10,032	0.03	Standard resin	Castable	Bio compatible	Flexible	Clear	[126]
	DWS’s XPRO S	300 × 300 × 300	27,000	0.01	Standard resin	Castable	Bio compatible	Flexible	Clear	[156]
	UnionTech’s RSPro 800	800 × 800 × 550	352,000	0.07–0.25	Standard resin	Castable	Bio compatible	Flexible	Clear	[157]
	3D Systems’ ProX 950	1500 × 750 × 550	618,750	0.01	Standard resin	Castable	Bio compatible	Flexible	Clear	[158]

**Table 2:** A general classification of available materials (commercial and laboratory-grade) for FDM, SLS, and SLA printing.

AM Technique	Material		
FDM	Thermoplastic filament	Semi-crystalline	PEEK
			PVDF
			PP
			PLA
			TPU
			TPE
			PPS
			PCL
			PLGA
			PEVA
			PA6
			PA12
			POM
			PET
		Amorphous	PEI
			PAI
			PPSU
			PC
			PVA
			HIPS
			PEKK
			ASA
			ABS
			PMMA
			PS
SLS	Thermoplastic powder	Semi-crystalline	PA12
			PA11
			PA6
			PET
			PLA
			PCL
			TPU
			POM
			PEEK
			PEK
			PEKK
		Amorphous	PC
			PMMA
			PS
			PI
			PSU
			PES
			PVA
SLA	Resins	Polyester	PPF
			PLA
			PCL
			PCL/PEG/Chitosan
		Polycarbonate	PTMC
			PTMC/Gelatin
			Trimethylolpropane Carbonate
		Polyether	PEG
			PEG/Chitosan
			PEO/PEG
			Poly tetrahydrofuran ether

**Table 3:** Melting temperature and glass transition temperature of a few common thermoplastic filaments for FDM.

	Material	T_m_ (°C)	T_g_ (°C)	Printing Temperature (°C)	Temperature of Degradation (°C)	Ref.
Thermoplastic Filament	ABS	-	105	230–250	380–430	[189,190]
	PLA	150	-	200–235	300–400	[190,191]
	PET	255	75	160–210	350–480	[190,191]
	PP	165	−10	230–260	300–500	[191,192]
	PA6	215	46	419.8	220–270	[189,193]

**Table 4:** Melting temperature, onset melting temperature, crystallization temperature, and sintering window of a few common thermoplastic powders for SLS.

	Material	T_m_	T_m_, Onset	T_c_, Onset	Sintering Window	Ref.
Thermoplastic powder	PA12	185.6	178.0	158.6	19.4	[108]
	PA11	202.9	189.2	168.3	20.9	[194]
	TPU	144.4	122.2	123.9	1.7	[194]
	PC	167.1	157.0	121.9	35.1	[195]
	PP	182.3	177.1	151.3	19.5	[196]

**Table 5:** A summary of the tensile properties of a few common print materials for all three, FDM, SLS, and SLA, printing techniques. Information such as print setting, test standard method, print orientation, tensile modulus, tensile strength, and elongation at break are provided.

Polymer		Supplier	Print Setting	Test Standard	Print Orientation	Tensile Modulus (MPa)	Tensile Strength (MPa)	Elongation (%)	Ref.
Thermoplastic Filament	PLA	3D Systems	100% infill Layer thickness 0.2 mm	ASTM D638	XYZ	1538	38.7	-	[212]
					YXZ	1246	31.1	-	
					XYZ	1350	33.6	-	
	ABS	Qimei Stock, China	100% infill	ASTM D638	XYZ	1200	37	-	[210]
	PEEK	Arevo Labs	100% infill Bed temperature 230 °C	ASTM D638	XYZ	2871	71.36	5.01	[211]
	PC	Stratasys, USA	100% infill	ASTM D638	XYZ	2410	54.6	4.22	[225]
	PP		100% infill Nozzle temperature 165 °C	DIN 53504-S3a	XYZ 0°	1230	34.3	-	[226]
					XYZ 45°	1000	32.0	-	
					XYZ 90°	1050	33.0	-	
ThermoplasticPowder	PA-12	Sinterit	Laser thickness 0.175 mm	ISO 527	ZXY 0°	864 ± 72	42.5 ± 3.1	13.1 ± 2.3	[227]
					ZXY 30°	690 ± 143	28.1 ± 8.4	6.7 ± 1.6	
					ZXY 45°	613 ± 27	16.0 ± 2.3	2.7 ± 0.3	
					ZXY 60°	694 ± 32	25.6 ± 8.9	8.4 ± 5.7	
					ZXY 90°	426 ± 150	17.1 ± 10.0	6.0 ± 3.4	
		Duraform	Layer thickness 0.1 mm Part bed temperature 175 °C Laser power 38 W	ISO 527-1	ZXY	1675 ± 41	47.6 ± 1.5	6.6 ± 0.7	[176]
					YXZ	1610 ± 61	40.6 ± 3.4	3.7 ± 0.6	
		Orgasol IS	Layer thickness 0.1 mm Part bed temperature 164 °C Laser power 48 W	ISO 527-1	ZXY	1700 ± 25	54.7 ± 0.7	12 ± 0.4	[176]
					YXZ	1580 ± 21	29.3 ± 3.6	1.9 ± 0.3	
		EOSINT	Laser power 3.33 W Powder bed temperature 140 °C			205.0 ± 29.3	57.7 ± 10.3	11.5 ± 1.3	[228]
	PA-11		Building chamber temp 157 °C Powder bed temperature 177 °C Layer thickness 0.3 mm	ISO 527-2			7.1 ± 0.5	5.9 ± 0.5	[177]
	PP		Powder bed temperature 150 °C Layer thickness 0.12 mm			-	27.9	-	[196]
		Trial Corporation	Powder bed temperature 150 °C Laser power 13.75 W Layer thickness 0.15 mm	ISO 527-2	ZYX	599.1 ± 14.1	19.9 ± 3.5	122.25	[229]
	PA 6	Mazzafero Tecnopolímeros S.A.	Powder bed temperature 120 °C Laser power 2.34 W			166.6 ± 77.8	62.4 ± 16.0	10.9 ± 3.7	[228]
	PC	HRPS	Laser power 13.5 W Layer thickness 0.15 mm Bed temperature 100 °C	ISO527-2	ZYX	40.12	1.1	5.05	[230]
Resin	PR 48	Autodesk, USA	Layer thickness 50 µm Print resolution		ZXY 0°	723	-	-	[219]
					ZXY 45°	350	-	-	
					ZXY 90°	376	-	-	
					ZXY 0°	901.4	-	-	
					ZXY 45°	667.1	-	-	
					ZXY 90°	182.2	-	-	
	Clear V4	Formlabs	Layer thickness 50 µm	ISO 527	Mean 0,15°,30°,45°,60°,75°,90°	2298	60.8	8.05	[217]
	Watershed 11122	DSM Somos	Layer thickness 0.175 mm Laser power 2.5 W Laser scanning speed 3200 mm/s	ASTM D638	XYZ 0°	37.75 ± 1.82	3.45 ± 0.11	11.67 ± 4.97	[221]
					XYZ 45°	43.25 ± 0.98	3.51 ± 0.03	7.60 ± 3.48	
					XYZ 90°/YXZ	38.24 ± 2.22	3.26 ± 0.08	8.53 ± 4.29	
					XZY 0°	46.07 ± 0.99	3.54 ± 0.07	9.27 ± 1.10	
					XZY 45°	47.70 ± 0.52	3.65 ± 0.02	9.00 ± 3.57	
					XZY 90°/YZX	45.72 ± 0.48	3.50 ± 0.05	6.60 ± 0.30	
	Monomer: EGPEA Crosslinker: 1,6-hexanediol diacrylate Photoinitiator: 2-Benzyl-2-(dimethylamino)-4′-morpholinobutyrophenone		Monomer 1, crosslinker 0.4 Monomer 1, crosslinker 1.0	ASTM D638		18.026 ± 0.302	1.861 ± 0.435	0.106 ± 0.025	[220]
						36.586 ± 1.210	2.243 ± 0.709	0.062 ± 0.021	

**Table 6:** A summary of the flexural properties of a few common print materials for all three, FDM, SLS, and SLA, printing techniques. Information such as print setting, print orientation, flexural modulus, flexural strength, and elongation at break are provided.

Polymer		Supplier	Print Settings	Sample Orientation	Flexural Modulus (MPa)	Flexural Strength (MPa)	Elongation (%)	Ref.
Thermoplastic Filament	ABS		100% infill	XYZ	1750	60	3	[231]
	PLA		100% infill Bed temperature 230 °C Nozzle temperature 210 °C	XYZ 0°	3187	102.203	10.6	[209]
				XYZ 45°	2985	90.649	7.8	
				XYZ 90°	3000	86.136	4.5	
	PEEK	Arevo Labs	100% infill Bed temperature 230 °C Nozzle temperature 340 °C	XYZ 0°	1972	114	10.6	[211]
				XYZ 90°	1954	83.59	5.81	
				XYZ 0° and 90°	2146	88.70	6.58	
ThermoplasticPowder	PA-12	Duraform, 3D Systems			546 ± 28	86 ± 5	11 ± 0.5	[232]
	PC		Laser power 13.5 W Layer thickness 0.15 mm Bed temperature 100 °C		93.83	2.48	-	[230]
	PA 2200	EOS	Laser power 25 W Building chamber temperature 170 °C	XZY 0°	551.24 + −5.6	59.23 + −4.1	4.9 + −0.74 mm	
				XZY 45°	433.05 + −61.4	46.25 + −6.4	4.96 + −0.56 mm	
				XZY 90°	345.39 + −41.5	19.89 + −2.8	3.28 + −1.51 mm	
Resin	Freeprint splint	DETAX GmbH			-	19.5 ± 2.5	-	[233]
	LuxaPrint Ortho Plus	DMG GmbH			-	39.3 ± 2.0	-	
	Nextdent Ortho Clear	Vertex-Dental B.V.			-	91.3 ± 5.9	-	
	Dental SG resin	Formlabs	Layer thickness 0.05 mm	ZXY 0°	1654.35 ± 152.27	117.48 ± 12.39	-	[88]
				ZXY 45°	1467.56 ± 89.36	130.73 ± 5.12	-	
				ZXY 90°	1456.73 ± 149.83	135.69 ± 5.93	-	

**Table 7:** A comparative overview of FDM, SLS, and SLA printing processes. The representation is created to provide a guideline only.

	Fused Deposition Modelling (FDM)	Selective Laser Sintering (SLS)	Stereolithography (SLA)
Operational principal	Material extrusion	Laser sintering	UV curing
Resolution	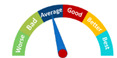	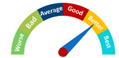	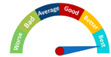
Accuracy	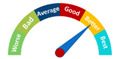	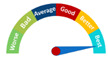	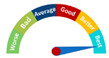
Surface finish	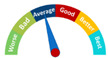	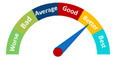	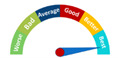
Design complexity	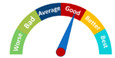	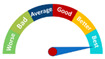	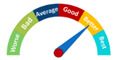
Ease of use	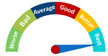	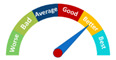	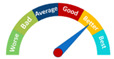
Printing time	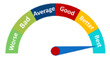	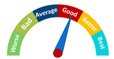	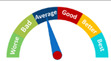
Advantages	Fast printing process Low part production cost Wide variety of materials are required	Functional parts Design freedom No support structures is required	High-resolution parts can be achieved Wide range of functional applications High accuracy
Limitations	Poor surface finish Support structures are required	Rough surface finish Lengthy printing time	Limited materials High maintenance cost is required

## Abbreviations

AM	Additive Manufacturing
CAD	Computer-Aided Design
ASTM	American Society for Testing and Materials
FDM	Fused Deposition Modelling
LOM	Laminated Object Manufacturing
WAAM	Wire and Arc Additive Manufacturing
EBF3	Electron Beam Free Form Fabrication
SLS	Selective Laser Sintering
EBM	Electron Beam Melting
SLM	Selective Laser Melting
LMD	Laser Metal Deposition
SLA	Stereolithography
MJ	Material Jetting
STL	Standard Tessellation Language
FFF	Fused Filament Fabrication
PLA	Polylactic Acid
SLA	Selective Laser Sintering
PP	Polypropylene
TPU	Thermoplastic Polyurethane
TPE	Thermoplastic Elastomer
ABS	Acrylonitrile Butadiene Styrene
TGA	Thermogravimetric Analysis
PA	Polyamide
PEEK	Polyether Ether Ketone
TGA	Thermogravimetric Analysis
PCL	Polycaprolactone
PLGA	Polylactic Glycolic Acid
PEI	Polyethylenimine
PEKK	Polyetherketoneketone
ASA	Acrylonitrile Styrene Acrylate
PMMA	Polymethyl Methacrylate
PS	Polystyrene
PET	polyethylene Terephthalate
PES	Polyethersulfone
PVA	Polyvinyl Alcohol
PPF	Polypropylene Fumarate
PTMC	Polytrimethylene Carbonate
PEG	Polyethylene Glycol
DSC	Differential Scanning Calorimetry
CIPs	Carbonyl Iron Powders
DLP	Direct Laser Processing
μCLIP	Micro-Continuous Liquid Interface Production
DIW	Direct Ink Write
